# Resources, attitudes and culture: an understanding of the factors that influence the functioning of accountability mechanisms in primary health care settings

**DOI:** 10.1186/1472-6963-13-320

**Published:** 2013-08-16

**Authors:** Susan M Cleary, Sassy Molyneux, Lucy Gilson

**Affiliations:** 1 Health Economics Unit, University of Cape Town, Cape Town, South Africa; 2 KEMRI-Wellcome Trust Research Programme, Kilifi, Kenya; 3 Health Policy and Systems Division, University of Cape Town, Cape Town, South Africa; 4 Department of Global Health and Development, London School of Hygiene & Tropical Medicine, London, UK

**Keywords:** Accountability, Governance, Primary health care, Low and middle income settings

## Abstract

**Background:**

District level health system governance is recognised as an important but challenging element of health system development in low and middle-income countries. Accountability is a more recent focus in health system debates. Accountability mechanisms are governance tools that seek to regulate answerability between the health system and the community (external accountability) and/or between different levels of the health system (bureaucratic accountability). External accountability has attracted significant attention in recent years, but bureaucratic accountability mechanisms, and the interactions between the two forms of accountability, have been relatively neglected. This is an important gap given that webs of accountability relationships exist within every health system. There is a need to strike a balance between achieving accountability upwards within the health system (for example through information reporting arrangements) while at the same time allowing for the local level innovation that could improve quality of care and patient responsiveness.

**Methods:**

Using a descriptive literature review, this paper examines the factors that influence the functioning of accountability mechanisms and relationships within the district health system, and draws out the implications for responsiveness to patients and communities. We also seek to understand the practices that might strengthen accountability in ways that improve responsiveness – of the health system to citizens’ needs and rights, and of providers to patients.

**Results:**

The review highlights the ways in which bureaucratic accountability mechanisms often constrain the functioning of external accountability mechanisms. For example, meeting the expectations of relatively powerful managers further up the system may crowd out efforts to respond to citizens and patients. Organisational cultures characterized by supervision and management systems focused on compliance to centrally defined outputs and targets can constrain front line managers and providers from responding to patient and population priorities.

**Conclusion:**

Findings suggest that it is important to limit the potential negative impacts on responsiveness of new bureaucratic accountability mechanisms, and identify how these or other interventions might leverage the shifts in organizational culture necessary to encourage innovation and patient-centered care.

## Background

District level health system governance is recognised as an important but challenging element of health system development in low and middle-income countries [1]. Accountability is a more recent focus in health system debates [2]. Accountability mechanisms are governance tools which seek to regulate answerability between the health system and / or citizens and between different levels of the health system [3]. In addition to answerability, accountability has an element of enforcement, which raises the question of “how to avoid performance becoming conformance with targets?” (p. 261) [4]. Ideally, all accountability mechanisms would be designed to achieve health system goals (such as promoting access to quality care), but in practice different forms of accountability may receive higher priority than others and may have a different focus [5]. An important broad distinction is between ‘external’ or community accountability mechanisms which may be used by non-state actors to hold public sector power-holders to account, and ‘internal’ or bureaucratic mechanisms that are comprised of the institutional oversights, checks and balances internal to the public sector [2]. External (or community) accountability has attracted significant attention in recent years [6], but internal (or bureaucratic) accountability mechanisms, and the interactions between the two forms of accountability, have been relatively neglected.

The Alma-Ata declaration on primary health care [7] provided a conceptualization of how services at the first level of care should be organized, delivered and managed within decentralized health systems [8]. In this conceptualization, decision-space – i.e. decision-making authority over planning, budgeting, managing and monitoring of activities [9]- would be transferred from the national level to the local level [10]. The declaration also emphasized the importance of involving citizens in health care priority setting [8]. It was argued that increased “decision-space” at lower levels of the health system, together with citizen involvement in priority setting, might enhance the responsiveness of the system to the varying needs of clients and citizens (given resource constraints). In defining responsiveness in this way, we recognise that different actors would understand responsiveness differently and that it would be possible to provide clinically appropriate care (following protocol) without being responsive to community members’ needs and priorities. However, many would argue for the importance of community members having an opportunity to voice their views and concerns, and have these issues responded to, in addition to clinically appropriate care.

More recently it has been argued that appropriate decision-space needs to be complemented by governance approaches that enable and sustain responsiveness, including by promoting system learning and accountability [11]. However, despite its importance, governance and accountability is a neglected area within health policy and systems research [1]. This is an important gap given the need, at district level, to strike a balance between: 1) achieving accountability upwards within the health system for example through centrally-led budgeting, planning and reporting arrangements that offer system-wide guidance and promote system level equity goals [12]; and 2) allowing for the local level innovation that could improve quality of care and responsiveness to patients and citizens.

Brinkerhoff and Bossert [13] offer an approach to conceptualizing governance that focuses on the micro level of relationships among system actors, nested within wider organizational and system settings. They describe health governance as being about putting in place effective rules that “condition the extent to which the various actors involved fulfill their roles and responsibilities and interact with each other, to achieve public purpose” (p3). Other institutional influences over interactions among actors, and their governance consequences, include the norms and values that confer responsibilities and rights, whether formal or informal. Brinkerhoff and Bossert [13] argue that when these interactions work well, they ensure accountability to beneficiaries and the broader public; a policy process that allows negotiation and compromise among different actors, and effective policy implementation.

Using a descriptive literature review, this paper reviews empirical literature about accountability mechanisms in the district health system from low and middle-income countries (LMICs) with a specific focus on public sector delivery of care. We aim to unpack the factors influencing how mechanisms work and the link between accountability mechanisms, and patient and citizen responsiveness. We are also interested in the interactions between mechanisms, including between the different bureaucratic mechanisms and between bureaucratic and community or external mechanisms.

## Methods

We searched the available literature for empirical papers on topics related to accountability in health services in LMICs, as defined by OECD. An initial set of published and grey literature for review was identified based on previous experience, through reviewing the reference lists of three published review articles [6,14,15] and through retrieving relevant papers. While many of the papers that were included in this initial literature examined external accountability mechanisms, there were few papers that evaluated bureaucratic accountability mechanisms, and there was also limited evidence regarding how the bureaucratic and external accountability mechanisms interacted with each other. Additional searches of published literature were therefore conducted in PubMed using combinations of the following key words: “target”, “target-setting”, “management”, “planning”, “budget”, “budgeting” and “accountability” together with “primary health care”, “district”, “developing” and “low middle income”. The titles and abstracts of papers meeting these search terms were reviewed and the full versions of potential papers were read to decide on final inclusion. The final set of papers was restricted to those that described or evaluated accountability mechanisms within the primary health care system (both within facilities and through the outreach efforts of community health workers and environmental health officers). Our focus was on public sector health services – in settings where private providers are included accountability mechanisms and structures would need to be different. We excluded non-English language papers.

### Conceptual framework

**Figure 1:**
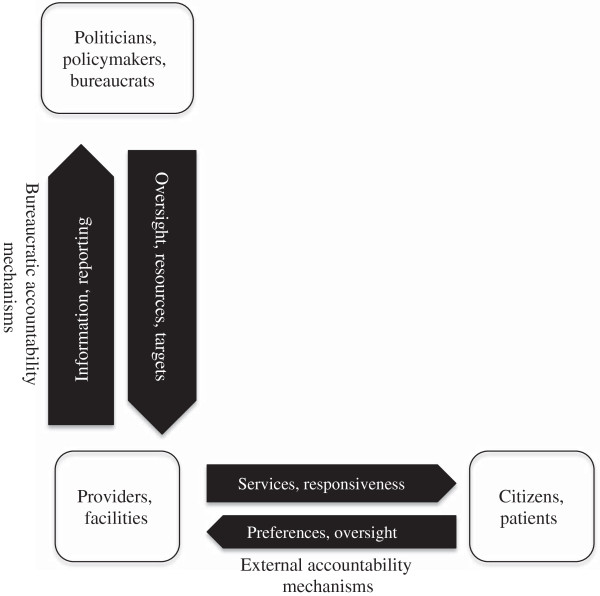
Framework of accountability mechanisms in health care.

Our review was guided by the conceptual framework presented in Figure 1, which is adapted from a health governance framework proposed by Brinkerhoff and Bossert [13]. Three sets of actors are depicted within the framework: (1) politicians, policymakers and bureaucrats located higher up within the health system hierarchy (including actors within health ministries, national and provincial departments of health and district health management teams); (2) providers operating within primary health care facilities or outreach workers within the district health system; and (3) patients and citizens. The arrows illustrate the relationships between these sets of actors, and the different nature of the arrows (in terms of size and transparency) suggests that the power and influence of the different categories of actors differs, with the state and providers tending to have more power, information and expertize than patients and citizens [13].

The vertical arrows linking providers to the state illustrate the relationships associated with bureaucratic accountability mechanisms, which function to promote answerability within the health system hierarchy. For example, the state may hold facilities and providers accountable through monitoring progress towards the achievement of health care targets, through setting budget and expenditure guidelines and through providing supervision and oversight [13]. Monitoring progress against targets, for example, is argued to be one way in which government can provide leadership, guidance and strategic direction for the health sector [16]. These targets are argued to provide benchmarks for the measurement of progress [16] while ensuring a minimal level of service delivery within settings and of equity between settings [17]. Health facility budgets are frequently determined at the provincial or national level, and reporting on spending can be used to hold providers accountable. However, services may be more responsive to local needs if expenditure categories and priorities are locally determined [10].

Bureaucratic accountability mechanisms also include a range of human resource management and supervision processes [13]. External supervision of facilities and providers, for example, is argued to be one way of linking the peripheral facility to the district, which may be important for maintaining performance and for motivating staff. Such supervision could include assisting with problem solving, reviewing facility records and checklists, and observing clinical practice [18]. Facility in-charges or managers may also be involved in performance monitoring, mentoring and disciplinary action within facilities, and may report on these processes to their managers at the district level [19].

Horizontal arrows illustrate the relationships between providers and patients and citizens and the associated external or community accountability processes, where citizens and patients may express their preferences about services, and may be involved in monitoring and supervising facilities and providers [13]. The functioning of external accountability processes requires providers to be responsive to citizen input, including through taking action to alter services in response to ideas or concerns raised by citizens [6]. External accountability mechanisms identified in the literature include involvement or participation of citizens in clinic and district health committees, the use of patient complaints procedures, provider report cards and patients’ rights charters [6].

## Results

The final set of literature for review included twenty-six empirical and five review articles [6,14,15,18,20]. These papers were reviewed for evidence about the functioning of accountability mechanisms in the district health system and the potential link to patient and citizen responsiveness. Details of the empirical papers are provided in Additional file 1. Papers were from Africa (10 countries), Asia (3 countries, and with one paper taking a regional perspective), and South America (4 countries). Many of the empirical papers used a case study design and the data collected were largely qualitative in nature. The main methods included observations, in-depth interviews, focus group discussions, and reviews of documents and meeting minutes. A number of papers introduced some form of a control or comparator through the selection of well performing and less well performing cases. One paper described a randomized field experiment [21].

Table 1 summarizes the key types of mechanisms that were identified within the reviewed empirical papers. Bureaucratic accountability mechanisms include 1) human resource management processes (including supervision, disciplinary action, mentoring); and 2) budgeting, planning and target setting processes. External mechanisms include a range of voice mechanisms such as 1) clinic and community committee involvement in the monitoring of providers and the setting of health care priorities; 2) provider report cards; 3) patient rights charters; and 4) complaints mechanisms. Some of the empirical papers described and/or evaluated more than one mechanism, such as the simultaneous use of budgeting and targeting approaches with human resource management approaches, or targeting approaches with external accountability mechanisms.

**Table 1:** Examples of accountability mechanisms and their functioning

**Type of mechanism**	**Example of functioning**	**Link to citizen responsiveness**
*Internal accountability*		
Human resource management	Regular performance appraisals between line managers and staff seek to evaluate the extent to which staff are meeting key performance areas. They also draw attention to the necessary competencies, behaviours and practices of staff needed to achieve agreed targets.	Indirect
Budgeting, planning, priority setting, target setting	Annual health care plans, based on an assessment of local needs, are used to guide resource allocation processes to districts and within districts; budget is allocated and targets are set; facilities report back to the district management on the extent to which targets have been met.	Indirect
*External accountability*		
Clinic committees	A forum for hearing local needs is provided. Information is exchanged and citizen or patient questions or complaints can be answered. Information on local needs is fed into priority setting processes.	Direct
Provider report cards	Patients rate the quality of care at facilities; information is provided to citizens about facility quality of care; information is provided to district management teams about where improvements/ commendations are merited.	Direct
Complaints boxes	Patients give input into service aspects needing improvement. Staff are sensitised to patient perspectives.	Direct

In the following sections, we report on key factors that influence the functioning of these accountability mechanisms. Results are presented first for external accountability processes, second for human resource management and external supervision processes and lastly for budgeting and target setting processes. The results are framed following three key themes that have emerged from the review as influencing functioning: resources (time, space and capacity), attitudes and perceptions of actors, and the values, beliefs and culture of the system. Clearly all three themes overlap and interlink, but we have reported each separately in order to facilitate understanding. The overlap is particularly marked in the case of attitudes and perceptions of actors versus values, beliefs and culture of the system. While system level values and organisational culture are related to individual beliefs and values, individual actors would not always share the values and beliefs advocated by the system.

### External accountability mechanisms

Two review articles [6,20] and several empirical papers (see Web Appendix 1) described the functioning of external accountability mechanisms. One review paper focused exclusively on the effectiveness of committees and the factors that influence their performance. This focus implied a review of the broader literature on community participation in committees [20]. By contrast, the second review article [6] focused on community accountability (which requires a deeper engagement than some models of community participation) and reviewed the literature for evidence of impact and the factors influencing impact for different mechanisms, including committees, provider report cards, complaints mechanisms, and patient rights charters.

#### ***Resources and capacity***

A key theme emerging from the articles related to the lack of resources allocated to external accountability initiatives as well as concerns about whether citizens have the capacity to hold providers to account [6,20,22-24]. Themes related to lack of capacity included the technical nature of health care [24-26], and lack of clarity in the roles and responsibilities of clinic committee members [6,20,27-29]. As observed by Molyneux et al [6], “regarding clarity in role, there remained particular uncertainty, confusion and sometimes conflict regarding extent of decision-making power” (p. 9). Articles also suggest the importance of training for providers and clinic committee members so that roles can be clarified and committee members can be empowered to manage their roles [20,25,26,30].

However, even with role clarity, low education levels continued to be a barrier [28,31]. In a Kenyan case study, a district manager suggested that older illiterate committee members struggled to engage in the committee, particularly in the area of financial management. This lead to health workers “usurping” (from a citizen perspective) the roles of community members. On the other hand, health workers argued that they had to intervene because the health service holds them rather than volunteer committee members accountable for the completion of accounting records [28]. This finding - pointing to the potential mismatches between bureaucratic and external accountability mechanisms - was mirrored in findings that suggested that committees may be unable to influence the allocation of budgets to community priorities if national priorities (as identified in national targets for particular disease groups and nationally determined expenditure categories, for example) take precedence over local processes [24,26,28,29,32]. In a Tanzanian case study, one member of the Council Health Management Team (the district management team that works with clinic committees to determine district priorities) explained that:

“…the government usually requires us to do the things it considers important…At times the things prescribed by the government are not of any importance to the district. Even so, we include them in our plans because the government has decided that they should be carried out” [29] (p. 7).

#### ***Attitudes and perceptions of actors***

The reviewed literature suggests that external accountability mechanisms require trusting interpersonal relationships between providers and citizen representatives. Where relationships were poor [23,32], community members reported feeling scared to raise complaints about facilities or providers [24,25]. As explained by a patient respondent in a Colombian case study: “ ‘People are used to being treated badly and to being abused and do nothing about it.’…They are afraid that health care personnel will retaliate by providing low quality health care or by refusing to attend them personally” (p. 56) [24].

From the perspective of providers, resistance to citizen involvement in health facility monitoring and supervision was reportedly related to perceptions that community members were behaving like watchdogs – exercising control and power without offering support [28,33]. In a Kenyan case study, the authors concluded that this perspective could result in the relatively more powerful provider seeking to control and dominate the accountability process [23].

Functioning of external accountability mechanisms could also be hampered by a perceived history of unfulfilled needs where community members may have low expectations regarding their health care entitlements [21,34,35] leading to a perception that holding providers accountable is a waste of time [22,24,34]. As argued by Mosquera et al [24], low expectations can be overcome through a long-term commitment to build positive working relationships with community members. In their case study from rural Brazil, Tendler and Freedheim [34] illustrated that trust could be built if the health system shows that it is acting on citizen inputs, including taking appropriate disciplinary action against providers that citizens identified as shirking responsibilities.

#### ***Values, beliefs, culture***

Several empirical papers [24,25,35,36] and review articles [6,20] highlighted the potential mis-match between the values and beliefs of the health system and local communities relative to the democratic and participatory values that underpin external accountability mechanisms. McCoy et al [20] argue that bureaucratic health systems may be unwilling to relinquish power to citizen groups. Similarly, Molyneux et al [6], argue that the socio-cultural norms and structures that would promote community participation in accountability mechanisms are not always present. For example, in a Tanzanian case study, when the district management team was asked why citizens did not express their opinions about health service priorities, “the common response was that this was a new culture and a majority of the public was not aware of their rights” [29] (p. 10).

### Management and supervision

Findings in this section are drawn from two review articles [15,18], complemented by several empirical papers. Bosch-Capblanch et al [18] reviewed the literature on primary health care supervision in developing countries with a focus on defining the scope of supervision in policy documents and in practice, and assessing the evidence for the effects on health service performance. Dieleman et al [15] reviewed the human resources literature with the aim of describing how governance issues have influenced human resource policy development and identifying strategies that have been used to improve human resource policy implementation in LMICs.

#### ***Resources and capacity***

The key theme identified in this section was the connection between decision-space and management practices. In many of the study contexts, facility managers, external supervisors, or clinic committee members would be given responsibility for managing and monitoring providers without being given the tools to do the job [19,37,38]. For example, in The Gambia, findings from a large scale management strengthening project suggested that while the project succeeded in improving management skills and systems within the regional health teams (that were charged with supervising service delivery within the decentralized system), their effectiveness was limited by the policy and practice of central government and donors including centralized decision-making on issues of staff establishments and budgets [39].

In settings where managers had limited discretion, two key managerial practices were identified in the literature. Managers either took the route of encouraging better practice through supportive mentoring approaches [19], or they took the route of discouraging poor practice through expressing their dissatisfaction through authoritarian approaches (shouting, blaming, criticizing and micro-managing) [19,38]. This authoritarian style in turn could be related to the frustration experienced by managers about their insufficient decision-space and poor conditions of service [19].

Another argument was that facility managers reproduce the management styles of their superiors (this theme is further developed in the section on organisational culture below). The author of a rich Indian case study [38] suggested that less experienced managers were more likely to lean upon authoritarian approaches, involving “grilling” their health workers, instead of offering support and mentorship. This could be because of their lack of capacity and experience (managers are often health workers with no formal managerial training) but also because “working alongside” and offering support to workers was seen to be a futile effort by managers who did not have the capacity to solve many of the problems experienced by their staff. As explained by George [40], “by enquiring into problems that require cooperation from powerful actors that are beyond their control, supervisors only raise false expectations and expose the futility of their roles” (p 213).

#### ***Attitudes and perceptions of actors***

The relationship between health workers and managers was found to be a key factor influencing health worker motivation and responsive behaviour. Some health workers described managers as supportive, motherly, collaborative, and hard working. Such managers were seen as role models and inspired their staff to do their best. On the other hand, there were reports of unfair treatment by managers or by the system [19,38,41]. This included unpaid salaries, lack of promotion and/or lack of merit-based promotion, unfair allocation of privileges (particularly training), lack of role clarity and being blamed for quality of care or other problems that health workers perceived to be beyond their control.

Beyond unfair treatment was the perception of a lack of support and care from managers and the health system more generally. Health workers who perceived a lack of care reported that they would be more likely to take their frustrations out on their patients and in this manner uncaring attitudes were propagated through the system from managers to providers to patients [19].

#### ***Values, beliefs and culture***

Findings in this section suggest that while accountability processes might be operationalized within interpersonal relationships, these relationships in turn are governed by a series of beliefs that operate at the social or organisational level which structure ways of being and ways of interacting. A key theme here was the professional identity of the health worker. Evidence suggests that some providers enter their professions owing to a sense of calling - a desire to help those in need [19,37] and that inspiring health workers around the “mission” of care could be a powerful motivator [34]. As explained by a provider in a South African study: “…when I wake up in the morning I feel the need to go to work inside me, no one forces me to go to work. Knowing I serve the community makes me feel good inside” (p. 20). However, others report frustration in their working conditions and the system level barriers to doing their job [19,41]. In the same South African study [19] one provider comments: “For example, with me I just wake up in the morning with no motivation whatsoever because I know there is nothing that I will do because we do not have medicine” (p. 21).

Professional identity was also found to influence the functioning of external accountability processes where citizens were given the task of monitoring providers. Some of the literature suggested that providers felt that it was beneath their dignity to be monitored by members of the public [14], and in some settings this was argued to be demotivating with potentially negative implications for patient responsiveness [19,41]. On the other hand, if the system is designed to enhance the status of the provider then professional identity can be a powerful support to motivation and potentially to responsive provider behaviour. This was one finding from a study in Brazil where the nurse supervisors of community health workers gained status in the community and felt that they had more discretion to practice nursing as “a professional” because of the greater decision space that had been delegated [34].

The hierarchical organisational culture that commonly characterises public systems of health care provision was another theme [18,19,38]. There was a sense that managers engaged in grilling and lecturing their staff because this was how they were managed by their supervisors. Similarly, health workers were rude to patients because of the perceived unfair practices of their managers [19,38]. In this way, hierarchical attitudes are disseminated within the health care organisation. Hierarchical management styles were also linked to patronage and corruption. When those in positions of power use this power to serve individual ends they would be unlikely to implement systems where management must act as a role model or be answerable for actions taken [38].

While hierarchical management styles were sometimes associated with low health worker motivation, a team based organisational culture and trust in management was found to be beneficial for motivation [19,42]. In one Ghanaian study of environmental health officers, good informal working relationships, a shared understanding of and commitment to the mission, and a hands-on and supportive management style were seen to be protective of motivational levels despite other demotivating factors [42]. While good relations with communities and patients are also motivating [42], the authors of a South African case study of health worker motivation concluded that trust in the manager and trust in colleagues may be more important [19].

### Budgeting and target setting approaches

Very few papers were found that examined in any detail the functioning of targeting and budgeting accountability approaches. However, these mechanisms featured in the external accountability, management and supervision literature. This allows an examination of how these bureaucratic mechanisms might intersect and influence the functioning of the more local level processes of external accountability and facility management.

#### ***Resources and capacity***

As suggested in the section on external accountability, targets, expenditure categories, and budgets that are determined at the county, provincial or national level may pose a barrier to the functioning of external accountability approaches [24,26,28,29,32]. This finding was echoed in a systematic review in which the authors concluded that oversight mechanisms encourage accountability of providers to the state and not necessarily to citizens and patients [14]. In a case study from The Gambia, these bureaucratic accountability approaches were also argued to conflict with delegated managerial practices within decentralized systems, where planning and budgeting was still controlled centrally [39].

An Indian case study particularly illustrated how time consuming accountability processes based on reporting on targets could be [38]. In speaking of meetings where targets were discussed one health worker stated: “These meetings are simply a waste of time…Almost 10 days each month goes to these meetings. They should leave us alone so that we can do our work” (p. 211) [38]. The functioning of budgeting and target setting approaches within responsive health systems was also dependent on the availability of good local data to tailor targets to local health care needs and on the availability of adequate training of local officials that were charged with collecting the target information and of designing the budget plans [10,25,32].

#### ***Attitudes and perceptions of actors***

Given the amount of time taken to collect and compile information for reports, health workers in an Indian case study reported finding it demotivating if this information were not used to guide planning or responsiveness. In addition, workers reported that it was unfair to reduce the complexity of their working lives to numerical outputs, and that the targets themselves might be unfairly set [38]. According to one provider in this study: “They are not interested in the work you actually do, only in the reports you submit” (p. 211) [38]. In a Zambian case study, budgeting processes were somewhat farcical in that it was clear that the funds would not be deployed. “The prevailing perception on budgeting was to think of budgets to be ‘like a dream, and that not all dreams come true’” [25].

#### ***Values, beliefs and culture***

As mentioned, an Indian case study suggested that the practice of accountability through targets could be perceived by health workers to be unfair and demotivating [38]. This was not only because of the reductionism associated with targets, but also because this practice was perceived to allow the system to pay lip service to the idea of the provision of patient care, when often the service functioned to serve other purposes (including corruption and patronage) [38]. Similarly, in a case study from rural Nepal, Aitken [43] suggests the need to be cognizant of what she termed the official versus implicit rationale for the existence of health care bureaucracies. While on paper the service was argued to be about the provision of patient care, in practice the service was seen to exist to pay salaries to workers irrespective of whether any care was provided. This implicit rationale was understood and protected by providers and managers although not officially acknowledged. Accountability mechanisms were therefore argued by the author to be designed and implemented such that the main duty of staff was to be present at their posts; once there it was irrelevant whether they did any work [43].

Finally, in reflecting on an unsuccessful attempt to alter budgeting and resource allocation approaches within a newly decentralized system in Pakistan, Green and colleagues [10] suggest that while their project focused on the lack of appropriate budgeting skills as well as the lack of local information on health needs (i.e. issues of capacity and resources), insufficient attention was paid to the decision-making culture. “The culture of centralized decision-making and an attendant procedurally driven bureaucracy, coupled with the frequent transfer of staff, means that decentralization both challenges the organizational and management culture and is in fact high risk” (p. 1032).

## Discussion

**Figure 2:**
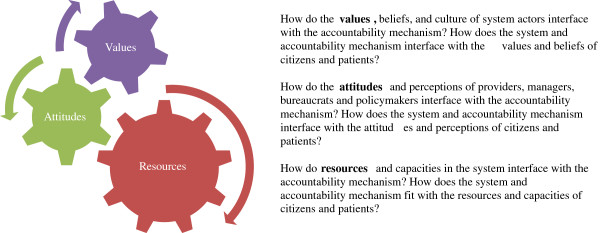
Factors influencing the functioning of accountability mechanisms.

In recent years, there has been increased interest in the potential for accountability mechanisms to improve the performance of health systems. This paper has undertaken a review of empirical studies that include an analysis of the functioning of such mechanisms within public primary care facilities and districts in low and middle-income countries. We aimed to unpack the factors that impact on functioning and have sought to draw a link to responsiveness. The literature review identified three sets of factors influencing functioning: 1) the values, norms, institutions and culture of health care, versus citizens and patients; 2) the attitudes and perceptions of providers, managers, bureaucrats and policymakers versus citizens and patients; and 3) the resources and capacities of the health service versus citizens and patients. These themes have emerged as a result of this review work, and are summarized within Figure 2. The three wheels presented in the figure suggest the inter-relationships between these elements – where the values of the system are reflected in resource flows; resources and capacity impact on attitudes and perceptions; and attitudes and perceptions impact on the use of capacity and contribute to enforcing or changing values. In the figure, the wheel for resources is depicted as being larger than the other two wheels. This reflects the idea that adequate resources and capacity is a necessary but insufficient condition for functioning. Further, once resources are adequate, the other wheels would become more important for functioning and the relative importance of these factors would differ between contexts.

The interlinked nature of the wheels captures the idea that the design of accountability mechanisms needs to consider the current configurations of resources, attitudes and values within the system as a whole, within different levels of the system, and how these interface with each other. Similarly, the design of a new mechanism needs to pay attention to similar elements and interfaces within citizens and patients. For example, mechanisms that involve citizens in the monitoring of providers need to take account of whether local citizens are sufficiently resourced and capacitated to play this role; whether providers are open to this form of input from the public or whether they would resist out of a perception that community members are behaving like “watchdogs”; and whether local communities have a culture of voluntary participation in local activities such as health care. In addition, even if community members have been capacitated to hold providers to account, power differentials between citizens and providers will continue to pose a barrier to the effective functioning of external accountability mechanisms. This implies that particular care should be taken to ensure that bureaucratic mechanisms are complementary to health system goals of responsiveness to avoid the tendency for bureaucratic accountability to crowd out external accountability and responsiveness to patients and citizens.

Key questions that might be used to guide those thinking about the design of accountability mechanisms are listed in Figure 2. The literature cannot answer the question of which combinations of accountability mechanisms may work best in which contexts. However, it does suggest that while resources and capacity are an obvious necessary condition of functionality, interpersonal relationships and organisational culture within health care are important to consider if patient and citizen responsiveness is to be improved. The link between organisational culture, relationships and accountability processes is clearly a key consideration in any intervention and context.

Ours is not the first review of accountability processes in health care. An earlier review by Berlan and Shiffman [14] focused on health provider accountability to consumers and grouped themes from the literature into health system factors (such as oversight mechanisms, revenue source, and nature of competition) and social factors (consumer power and provider norms). Our approach which unpacks functioning within different types of mechanisms (external and bureaucratic) offers additional insights into what works in different contexts and for different mechanisms. The other reviews included in this paper focused on a narrower subset of the governance and accountability literature including community participation in clinic committees [20], community accountability mechanisms [6], governance approaches in human resource policy design [15] and the effectiveness of external supervision of health facilities [18]. By including a broader evidence base, we are able to offer a more comprehensive perspective on the functioning of different mechanisms in complex health systems, particularly in terms of the interactions between the different bureaucratic mechanisms (e.g. budgeting versus management) and the interactions between bureaucratic and external accountability mechanisms. We have however excluded the growing body of evidence on performance-based pay, although the existing pay for performance evidence suggests that our review would be of interest and of relevance to those debates [44]. In addition, while our search may not have been broad enough to identify all of the relevant literature, we have been able to unpack a range of influences within three key themes, which could usefully be applied in future work.

The empirical papers included in this review mainly adopted a case study design and qualitative data collection techniques. They therefore offered “thick” descriptions of why different mechanisms may or may not work within different contexts. In synthesising this literature, we seek to offer analytic generalisations and to draw out key lessens for policy development. However, given the limited amount of evidence regarding bureaucratic mechanisms, it is clear that more research is needed about the functioning of these mechanisms, how they interact with each other and how they interact with external accountability mechanisms and promote or hinder patient and citizen responsiveness. In addition, given the current evidence it has not been possible to tease out the impacts on functioning according to whether mechanisms aimed to promote accountability as answerability or as enforceability.

## Conclusion

Accountability mechanisms could be key tools for ensuring the answerability of public primary health care facilities to central bureaucracies through the district health system, while at the same time providing the local decision-space that could increase citizen and patient responsiveness. While evidence is limited, this review suggests that the design of accountability mechanisms should pay attention to the attitudes and perceptions of actors, values of the system and resources, and that different (combinations of) mechanisms would be needed for different contexts.

## Competing interests

The authors declare that they have no competing interests.

## Authors’ contributions

LG conceived of idea for the article and identified an initial set of literature. SM reviewed the initial literature and designed the first conceptual framework for the review. SC reviewed the initial literature, identified further literature, and designed the final conceptual framework. SC wrote the first version of the article and all authors read and commented on this and subsequent versions. All authors read and approved the final manuscript.

## Pre-publication history

The pre-publication history for this paper can be accessed here:

http://www.biomedcentral.com/1472-6963/13/320/prepub

## Supplementary Material

Additional file 1 Empirical papers included in the review. Click here for file
