# Supplementary material for: Resources, attitudes and culture: an understanding of the factors that influence the functioning of accountability mechanisms in primary health care settings
Source: BMC Health Serv Res. 2013 Aug 16;13:320. doi: 10.1186/1472-6963-13-320 (PMC3844434; doi:10.1186/1472-6963-13-320)
Supplement: Additional file 1 — Empirical papers included in the review. [file 1472-6963-13-320-S1.docx]

Additional file 1: Empirical papers included in the review

|  | Accountability mechanism(s) | Setting and context | Study design | Link to responsiveness | Reference |
| --- | --- | --- | --- | --- | --- |
|  |  |  |  |  |  |
|  | Strengthening budgeting and resource allocation processes in a decentralized system | Province of Balochistan, Pakistan | Case study | Indirect | [[8](#_ENREF_8)] |
|  | Community participation in clinic committees | 3 Zimbabwean districts | Cross-sectional community surveys, key informant interviews, focus group discussions, secondary data analysis | Direct | [[44](#_ENREF_44)] |
|  | Community participation in clinic committees | Orumba South Local Government Area, Nigeria | Rapid appraisal of 25 clinic committees; in-depth case studies of 2 clinic committees | Direct | [[29](#_ENREF_29)] |
|  | Community participation in ward and village development committees | 2 Tanzanian districts | Multiple case embedded exploratory study using focus group discussions | Direct | [[21](#_ENREF_21)] |
|  | Community participation in 18 varying initiatives | Various settings in Asia | Document review of World Bank supported health sector reform programmes in Asia | Direct | [[34](#_ENREF_34)] |
|  | Community participation, budgeting processes | 8 “local health systems” in Brazil and Chile (4 in each country; half rural) | In depth case studies | Direct | [[15](#_ENREF_15)] |
|  | Community participation in clinic committees and through information sharing at facilities | 2 districts in Kenya | Case studies including interviews, record reviews, outpatient exit interviews, focus group discussions | Direct | [[32](#_ENREF_32)] |
|  | Community participation in clinic committees | 1 Kenyan district | Case study | Direct | [[31](#_ENREF_31)] |
|  | Community participation in clinic committees | Ulanga District, Tanzania | Rapid appraisal of 19 clinic committees; in-depth case studies of 2 clinic committees | Direct | [[22](#_ENREF_22)] |
|  | Community participation in budgeting and planning | 4 Zambian districts | Policy analysis including interviews, group discussions, participatory research action techniques, document reviews. | Direct | [[24](#_ENREF_24)] |
|  | Community participation | Oaxaca State, Mexico | Case study including semi-structured interviews, facility observations at 40 health centres | Direct | [[35](#_ENREF_35)] |
|  | Community participation in user associations and through customer service offices | Cali, Colombia | Case study including focus group discussions, semi-structured interviews and structured questionnaires | Direct | [[23](#_ENREF_23)] |
|  | Community participation in human resource monitoring through provider report cards | 9 districts in Uganda | Randomized field experiment | Direct | [[20](#_ENREF_20)] |
|  | Community participation in monitoring a cost recovery system; external supervision of facilities and providers | Tillaberi District, Niger | Case study of 11 health facilities | Direct | [[30](#_ENREF_30)] |
|  | Community participation in health facility monitoring | Tororo and Busia Districts, Uganda | 35 in-depth interviews at 4 health units | Direct | [[40](#_ENREF_40)] |
|  | Internal supervisors and community monitoring | Ceara State, Brazil | Case study | Direct and indirect | [[33](#_ENREF_33)] |
|  | Supervision and motivation | Rural setting in Nepal | Case study | Indirect | [[42](#_ENREF_42)] |
|  | Intrinsic versus extrinsic motivation to work | Arusha region, Tanzania | Observations of 80 clinicians in 39 facilities using vignettes | Indirect | [[36](#_ENREF_36)] |
|  | Supervision and disciplinary action; reporting on targets | Koppal, Northern Karnataka, India | Participant observations, semi-structured interviews, qualitative responses from a survey. | Indirect | [[37](#_ENREF_37)] |
|  | Supervision and management | Accra and Kumasi cities, Ghana | Case study | Indirect | [[41](#_ENREF_41)] |
|  | Supervision and management | District in North-East South Africa | In-depth inquiry in 4 primary health facilities using rapid ethnographic approaches | Indirect | [[17](#_ENREF_17)] |
|  | Community participation, budgeting and management | Mbarali District, Tanzania | Realist evaluation using interviews, non-participant observation and document reviews | Direct and indirect | [[28](#_ENREF_28)] |
|  | Community participation, budgeting and management | Mbarali District, Tanzania | Qualitative case study including key informant interviews, non-participant observation and document reviews | Direct and indirect | [[25](#_ENREF_25)] |
|  | Community participation, budgeting and management | Kwale and Tana River Districts, Coast Province, Kenya | Structured interviews with health worker in-charges and patients, in-depth interviews with district managers, health workers and health facility committee members | Direct and indirect | [[27](#_ENREF_27)] |
|  | Community participation and management strengthening | Lusaka, Zambia and Dar es Salaam, Tanzania | Document reviews, health worker interviews, focus group discussions, exit interviews with patients, community household survey. | Direct and indirect | [[26](#_ENREF_26)] |
|  | Management strengthening | 2 health regions in The Gambia | No specific details provided | Indirect | [[38](#_ENREF_38)] |
|  |  |  |  |  |  |
